# Supplementary material for: Bayesian networks to estimate prognosis in vascular cognitive impairment and small vessel disease: Integrated analyses of interdependent contributors to multiple outcomes
Source: J Cereb Blood Flow Metab. 2026 Oct 2:0271678X261496217. Online ahead of print. doi: 10.1177/0271678X261496217 (PMC13675423; doi:10.1177/0271678X261496217)
Supplement: Supplementary file 1 [file sj-docx-1-jcb-10.1177_0271678X261496217.docx]

**Supplemental Material**

**Supplemental Figures**

**Supplemental Figure S1.** Unconstrained BN structure learned without clinically informed hierarchical ordering

*This visualization shows the structure of the unrestricted BN, in which no clinically informed hierarchical ordering has been set. The arrows represent directed dependencies learned by the algorithm without restrictions on possible directions. Variables are grouped according to the same color layers (L0–L7) as in the constrained models, but the relationships shown are derived solely from the data. In this network, various connections arise that do not correspond to known or plausible clinical directions. For example, the vascular risk score appears as a parent variable of age and sex, even though this score is partly determined by age and sex, and there is an arrow from the baseline CDR to structural brain volumes, indicating reverse causality. The figure thus shows how, in the absence of clinical structural constraints, the model learns dependencies that are statistically possible but not interpretable in terms of content or causally implausible. This network therefore serves as a reference point.*

**Supplemental Tables**

**Supplemental Table S1:** Missing values per baseline variable

| **Variable** | **Network** | **n missing** | **% missing** |
| --- | --- | --- | --- |
| Age | Both | 0 | 0.0 |
| Sex | Both | 0 | 0.0 |
| Patient group | Both | 0 | 0.0 |
| Atherosclerotic cardiovascular disease history | Both | 0 | 0.0 |
| Cerebrovascular accident history | Both | 0 | 0.0 |
| Mini-Mental State Examination | Both | 3 | 0.5 |
| Baseline CDR | Both | 7 | 1.2 |
| Vascular risk score (SCORE2/SCORE2-OP) | Both | 12 | 2.1 |
| Brain/intracranial volume | Both | 17 | 3.0 |
| Starkstein depression score | Both | 24 | 4.2 |
| Small vessel disease score | Both | 54 | 9.5 |
| Hippocampus/intracranial volume | Both | 65 | 11.5 |
| Aβ40 | Biomarker-extended | 24 | 4.2 |
| Aβ42 | Biomarker-extended | 24 | 4.2 |
| GFAP | Biomarker-extended | 24 | 4.2 |
| NfL | Biomarker-extended | 24 | 4.2 |
| pTau181 | Biomarker-extended | 24 | 4.2 |
| Biomarker compound score 1 | Biomarker-extended | 58 | 10.2 |
| Biomarker compound score 2 | Biomarker-extended | 58 | 10.2 |
| Cerebral blood flow | Biomarker-extended | 99 | 17.5 |

*Missing values per baseline variable entered into the Bayesian networks, prior to imputation (N = 566). “Network” indicates whether the variable was included in both the base and the biomarker-extended network, or only in the biomarker-extended network. The outcome nodes (CDR increase, MACE) and the study discontinuation node contain no missing values and are therefore not listed; outcomes that were not observed are represented as an explicit state rather than as missing data. Cerebral blood flow was unusable in 99 participants: no gradable pCASL acquisition in 65 and failure at visual quality control in 34. Aβ = amyloid beta; CDR = Clinical Dementia Rating; GFAP = glial fibrillary acidic protein; MACE = major adverse cardiovascular events; NfL = neurofilament light chain; pTau181 = phosphorylated tau 181.*

**Supplemental Table S2**. Unadjusted and age- and sex-adjusted logistic regression associations between emerging plasma biomarkers and five-year cognitive decline (CDR increase) and major adverse cardiovascular events.

| **Outcome** | **Biomarker** | **N** | **OR (95% CI) unadjusted** | **OR (95% CI) adjusted age + sex** | **P unadjusted** | **P adjusted** |
| --- | --- | --- | --- | --- | --- | --- |
| CDR increase | Aβ40 | 315 | 1.01 (1.00, 1.03) | 1.01 (1.00, 1.02) | 0.02 | 0.13 |
| CDR increase | Aβ42 | 315 | 1.09 (0.95, 1.24) | 1.07 (0.94, 1.23) | 0.21 | 0.29 |
| CDR increase | BCS-1 | 304 | 2.47 (1.61, 3.77) | 2.23 (1.45, 3.44) | < 0.01 | < 0.01 |
| CDR increase | BCS-2 | 304 | 1.72 (1.23, 2.40) | 1.66 (1.16, 2.38) | < 0.01 | < 0.01 |
| CDR increase | CBF | 273 | 0.97 (0.95, 0.99) | 0.98 (0.95, 1.00) | < 0.01 | 0.06 |
| CDR increase | GFAP | 315 | 1.01 (1.01, 1.02) | 1.01 (1.00, 1.02) | < 0.01 | < 0.01 |
| CDR increase | NfL | 315 | 1.02 (1.00, 1.03) | 1.01 (1.00, 1.02) | 0.03 | 0.14 |
| CDR increase | pTau181 | 315 | 1.46 (1.15, 1.86) | 1.30 (1.02, 1.67) | < 0.01 | 0.04 |
| MACE | Aβ40 | 313 | 1.00 (0.99, 1.01) | 0.99 (0.98, 1.00) | 0.71 | 0.20 |
| MACE | Aβ42 | 313 | 0.99 (0.86, 1.13) | 0.98 (0.85, 1.12) | 0.83 | 0.72 |
| MACE | BCS-1 | 297 | 1.97 (1.33, 2.91) | 1.90 (1.27, 2.83) | < 0.01 | < 0.01 |
| MACE | BCS-2 | 297 | 1.73 (1.24, 2.42) | 1.66 (1.17, 2.34) | < 0.01 | < 0.01 |
| MACE | CBF | 265 | 0.99 (0.97, 1.02) | 1.00 (0.97, 1.03) | 0.54 | 0.97 |
| MACE | GFAP | 313 | 1.01 (1.00, 1.01) | 1.00 (1.00, 1.01) | 0.03 | 0.23 |
| MACE | NfL | 313 | 1.02 (1.01, 1.04) | 1.02 (1.00, 1.03) | < 0.01 | 0.02 |
| MACE | pTau181 | 313 | 1.01 (0.80, 1.29) | 0.86 (0.65, 1.15) | 0.90 | 0.31 |

*Complete-case analyses per biomarker, defined as an observed outcome and a non-missing biomarker value; sample sizes therefore differ between biomarkers. Missing baseline values were not imputed for these analyses. Adjusted models include age and sex, which are complete, so adjusted and unadjusted models have the same N. Aβ = amyloid beta; BCS = biomarker compound score; CBF = cerebral blood flow; CDR = Clinical Dementia Rating; CI = confidence interval; GFAP = glial fibrillary acidic protein; MACE = major adverse cardiovascular events; NfL = neurofilament light chain; OR = odds ratio; pTau181 = phosphorylated tau 181.*

**Supplemental Table S3**. Percentile-based bootstrap intervals for cognitive decline and major adverse cardiovascular events for a prototypical low-risk patient profile inferred from 200 resampled Bayesian networks

| **Scenario** | **Outcome** | **Category** | **Mean probability** | **Standard deviation** | **2.5%** | **97.5%** |
| --- | --- | --- | --- | --- | --- | --- |
| Age 58, Female sex, Reference, SVD-score = 0, MMSE = 29, Baseline CDR = 0 | CDR increase | No | 0.61 | 0.08 | 0.46 | 0.80 |
| Age 58, Female sex, Reference, SVD-score = 0, MMSE = 29, Baseline CDR = 0 | CDR increase | Study discontinuation | 0.27 | 0.05 | 0.15 | 0.38 |
| Age 58, Female sex, Reference, SVD-score = 0, MMSE = 29, Baseline CDR = 0 | CDR increase | Yes | 0.12 | 0.05 | 0.03 | 0.20 |
| Age 58, Female sex, Reference, SVD-score = 0, MMSE = 29, Baseline CDR = 0 | MACE | No | 0.60 | 0.08 | 0.44 | 0.76 |
| Age 58, Female sex, Reference, SVD-score = 0, MMSE = 29, Baseline CDR = 0 | MACE | Study discontinuation | 0.27 | 0.05 | 0.20 | 0.38 |
| Age 58, Female sex, Reference, SVD-score = 0, MMSE = 29, Baseline CDR = 0 | MACE | Yes | 0.12 | 0.03 | 0.23 | 0.26 |

**Supplementary Methods**

**MRI processing**

Processing of the brain MRI was performed using two automated pipelines. For each patient, manual segmentation of infarcts and other pathologies that potentially affect automatic tissue segmentation was performed by a neuroradiologist. Subsequently, the annotated infarcts and pathologies were manually segmented by trained students. In addition, an automated pipeline (Quantib Brain, Rotterdam, the Netherlands) was used to segment WMH based on FLAIR scans. A brain tissue segmentation method was applied to the 3D T1-weighted images. From these segmentations, volumes in milliliters (mL) of total brain gray matter (GM), white matter, cerebrospinal fluid, and WMH were computed.

**Plasma biomarkers**

Ethylenediaminetetraacetic acid (EDTA) plasma was obtained by venipuncture and, after centrifugation at 1,800×g for 10 minutes at room temperature, aliquoted (0.5 mL) into polypropylene tubes and stored at −80°C. Before analysis, samples were briefly thawed at room temperature and spun at 10,000×g for 10 minutes to remove residual debris that could interfere with the assays. Plasma Aβ42, Aβ40, GFAP, and NfL concentrations were quantified using the Simoa Neurology 4‑plex E kit (Quanterix, Billerica, MA, USA), and plasma pTau181 was measured with the Simoa pTau181 V2 kit (Quanterix) on the Simoa HD‑X platform. All measurements were performed in duplicate and followed the manufacturer’s protocol, including an automated 1:4 on‑board dilution step. The mean intra‑assay coefficients of variation (CVs) were 2.4% for Aβ40, 2.6% for Aβ42, 5.1% for GFAP, 4.3% for NfL, and 7.4% for pTau181, while inter‑assay CVs were 6.5% for Aβ40, 5.9% for Aβ42, 6.7% for GFAP, and 5.7% for NfL, based on three quality‑control samples across seven runs; for pTau181, the mean inter‑assay CV was 7.8%, calculated from two quality‑control samples measured in six runs. Two previously published Olink plasma biomarker compound scores (BCS), reflecting inflammation and coagulation processes, have also been added, which are described in more detail elsewhere (19,20).

**Cerebral blood flow**

Normal appearing gray matter CBF was measured with pCASL (multi-slice 2D echo-planar imaging [EPI] acquisition with background suppression; labeling duration = 1800 milliseconds; post-labeling delay = 1800 milliseconds; single-shot EPI readout; resolution = 3 × 3 ×7 mm3).2,12 pCASL data were processed using the automated Iris pipeline for CBF quantification . Quantification of pCASL data into CBF maps was based on a single- compartment model after the subtraction of labeled images from control images.12 To scale the signal intensities of the subtracted pCASL images to absolute CBF units, a separately acquired proton density weighted image was used. The quantification further included motion-correction of the raw pCASL data and partial volume correction (PVC). CBF was quantified in normal-appearing gray matter only. To obtain the normal-appearing gray matter mask for each participant, first a binary gray matter segmentation was obtained. Subsequently, PVC-uncorrected pCASL images of all participants were visually inspected. Images with suboptimal quality (i.e., motion artefacts, incomplete ASL-sequence, or labeling errors) and images with dominant vascular artefacts and little tissue perfusion signal were not used for further analyses. Baseline pCASL was unusable in 99 of 566 participants (17.5%): no gradable acquisition in 65 (11.5%) and failure at visual quality control in 34 (6.0%). By group: carotid occlusive disease 27/109 (24.8%), memory clinic–VCI 33/166 (19.9%), heart failure 26/162 (16.0%), reference 13/129 (10.1%).
